# A global coral-bleaching database, 1980–2020

**DOI:** 10.1038/s41597-022-01121-y

**Published:** 2022-01-20

**Authors:** Robert van Woesik, Chelsey Kratochwill

**Affiliations:** Institute for Global Ecology, Florida Institute of Technology, Melbourne, Florida 32901 United States of America

**Keywords:** Climate-change ecology, Marine biology

## Abstract

Coral reefs are the world’s most diverse marine ecosystems that provide resources and services that benefit millions of people globally. Yet, coral reefs have recently experienced an increase in the frequency and intensity of thermal-stress events that are causing coral bleaching. Coral bleaching is a result of the breakdown of the symbiosis between corals and their symbiotic microalgae, causing the loss of pigments and symbionts, giving corals a pale, bleached appearance. Bleaching can be temporary or fatal for corals, depending on the species, the geographic location, historical conditions, and on local and regional influences. Indeed, marine heat waves are the greatest threat to corals worldwide. Here we compile a Global Coral-Bleaching Database (GCBD) that encompasses 34,846 coral bleaching records from 14,405 sites in 93 countries, from 1980–2020. The GCBD provides vital information on the presence or absence of coral bleaching along with site exposure, distance to land, mean turbidity, cyclone frequency, and a suite of sea-surface temperature metrics at the times of survey.

## Abstract (summary)

**Table:** 

Measurement(s)	coral bleaching process
Technology Type(s)	field estimates
Factor Type(s)	sea surface temperature metrics
Sample Characteristic - Organism	Scleractinia
Sample Characteristic - Environment	marine benthic biome
Sample Characteristic - Location	global

Machine-accessible metadata file describing the reported data: 10.6084/m9.figshare.16958353

## Background & Summary

**Fig. 1:**
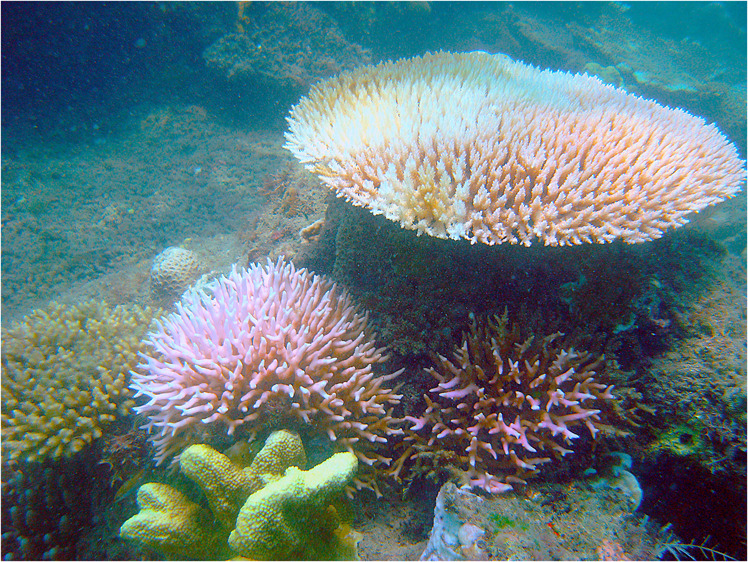
Bleached corals on the reefs of Bolinao, the Philippines, in 2005, during a thermal stress event. Photo R. van Woesik.

The ubiquity of reef-building corals stems from their capacity to support symbiotic unicellular dinoflagellates, from the family Symbiodiniaceae, within their tissues^1^. The symbionts photosynthesize and translocate photosynthates to the coral animals, and in return corals produce organic wastes upon which the symbionts thrive^2^. This mutually beneficial relationship between corals and their symbionts has allowed corals to thrive in shallow, tropical and subtropical localities and build coral reefs for millennia. Recently, however, this relationship has become dysfunctional during marine heat waves, when seawater temperatures are anomalously high^3,4^. This dysfunctionality leads to the paling of corals through loss of pigmentation or loss of symbionts — more commonly referred to as coral bleaching (Fig. 1)^3,5^. There is however considerable spatial and temporal variation in coral bleaching, depending on the intensity of thermal-stress events, geographic location^6^, the coral species^7^, historical conditions^8^, and on local and regional influences^9^. Here we were motivated to collate data on coral bleaching from around the globe, starting from 1980.

**Fig. 2:**
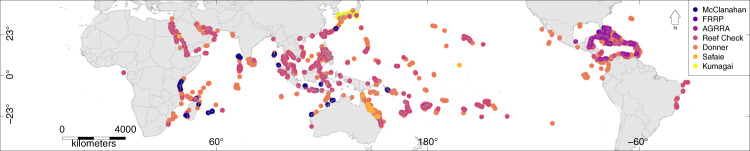
Study sites of the Global Coral Bleaching Database (GCBD) colored by data source.

Two databases have previously been compiled, one by ReefBase (4146 records) (http://www.reefbase.org), which was terminated around 2010, and the second by Donner *et al*.^10^ who collated 7429 data records on coral bleaching. Here we follow the previous database conventions to present a Global Coral-Bleaching Database (GCBD), obtained from seven data sources that encompasses 34,846 coral bleaching records from 14,405 sites in 93 countries, over 40 years, from 1980–2020 (Fig. 2). The database contains information on the presence and absence of coral bleaching—allowing comparative analyses and the determination of geographical bleaching thresholds—together with site exposure, distance to land, mean turbidity, cyclone frequency, and a suite of sea-surface temperature metrics at the times of survey.

## Methods

**Fig. 3:**
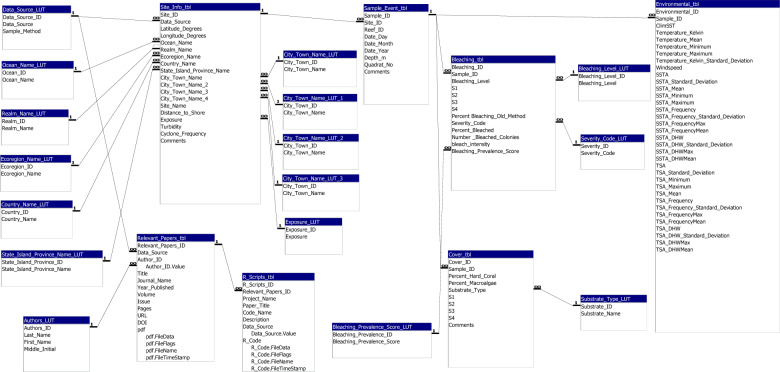
Schematic of the Global Coral Bleaching Database (GCBD) showing the relationships among the 20 tables.

The Global Coral Bleaching Database (GCBD) is available as a Microsoft Access database file and as a SQLite database file, the latter of which is directly accessible through R^11^. Examples of the R code that extracts data from the SQLite files ready for data analysis are provided in Table “R_Scripts_tbl”. Data in the GCBD are stored in 20 related tables (see Fig. 3 Schematic of the database structure). The static location data (latitudinal and longitudinal coordinates, distance to land, and exposure) are stored in the Table “Site_Info_tbl”. The primary geographical variable is a ‘site’ on a reef, recorded as latitude and longitude coordinates. A site can have multiple sampling events (i.e., multiple depths and/or multiple dates sampled), and these temporal events are stored separately in the Table “Sample_Event_tbl”. Data collected during these sampling events are stored in three related tables: “Coral Bleaching data tbl” (% bleaching), “Coral Cover data tbl” (% hard coral cover), and “Environmental data tbl”. Bleaching is an estimate of the number of bleached coral colonies relative to the number of colonies that are not bleached at a given site (i.e., site-wide bleaching). For any range estimates of coral bleaching, we took the mean value. Published works and any R code related to extracting or manipulating data are also stored in the R_Scripts_tbl and the Relevant_Works_tbl connected to the sampling event. Tables with enumerated lists are used to ensure integrity in naming conventions — such tables are denoted with “LUT”, where LUT stands for look-up-table.

### Normalization

If the site coordinates were not already in decimal degrees, they were converted to decimal degrees. The coordinates were entered into Google Earth and the location names, distance to land in meters, and exposure were determined for each site. Exposure was defined based on a site’s potential exposure to predominate winds, swell, and fetch (i.e., extent of open ocean). Sampling points that fell on land or were >1 km from any coral reef were removed. The Marine Ecoregions of the World (MEOW) shapefiles were used to determine the marine realm of each site^12^. Veron’s ecoregions shapefiles were used to determine the ecoregion of each site^13^. The Coral Reef Temperature Anomaly Database (CoRTAD version 6), which is a collection of sea surface temperature variables, were extracted for each sampling event^14^. CoRTAD values were only extracted for a sampling event if the coral bleaching data had a clearly defined month and year — where sampling events were missing a date, the 15^th^day of the month was used. Cyclone frequency and turbidity data were added for each site^15^. For turbidity, we used a 4-km resolution data from NASA’s (National Aeronautics and Space Administration’s) Earth Observing System Data and Information System (EOSDIS) Modis-Aqua satellite database. We acquired these data from mid-2002 through to December 2017 (https://oceandata.sci.gsfc.nasa.gov/MODIS-Aqua/Mapped/Monthly/4km/Kd_490/). Cyclone data were collected from International Best Track Archive for Climate Stewardship (IBTrACS; www.ncdc.noaa.gov/ibtracs/index.php?name=ibtracs-data) as spatial points and imported into R^11^. These data were subset into storm categories based on wind speed, according to the Saffir–Simpson scale^15^. A raster file for the spatial frequency of cyclones was made in Quantum Geographical Information Systems (QGIS) using the ‘heatmap’ function, with a radius matching the radius of damaging winds (>26 ms^−1^) for each cyclone category. These radii followed Moyer *et al*.^16^ and considered 50 yr of consistent sampling effort, between 1964 and 2014. Individual yearly raster files were summed to determine the number of cyclones per 9.2 km cell for the 50-year period. A raster file for the frequency of cyclones was created by interpolating wind speeds across all storm tracks using the inverse distance weighted interpolation in QGIS^15^. The Atlantic and Gulf Rapid Reef Assessment (AGRRA)^17^ and the Florida Reef Resilience Program (FRRP)^18^ had bleaching codes that were presented by transect instead of by site; these data were averaged and presented here at the site level. We did not include coral cover estimates for AGGRA and FRRP because both sampling strategies were designed to estimate coral populations at regional scales and not specifically to examine coral cover on reefs. Average depths (m) were used for the Donner *et al*.^10^ data that had ranges in depth.

**Fig. 4:**
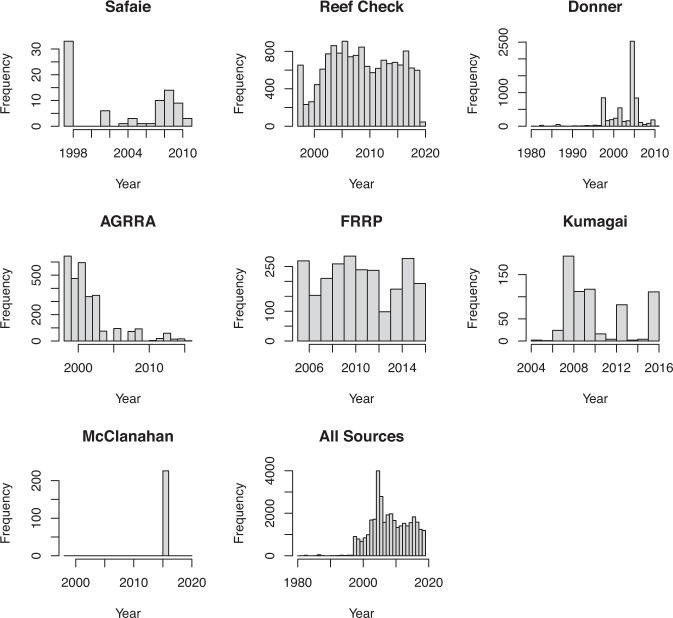
Datasets that were included in the Global Coral Bleaching Database (GCBD): Safaie *et al*.^21^, Reef Check (http://data.reefcheck.us/), Donner *et al*.^10^, AGRRA (https://www.agrra.org), FRRP: http://frrp.org/data/, Kumagai *et al*.^22^, McClanahan *et al*.^20^, and all surveys combined.

Datasets that were included in the GCBD included: (1) Reef Check (http://data.reefcheck.us/)^19^, (2) Donner *et al*.^10^, (3) McClanahan *et al*.^20^, (4) AGRRA (https://www.agrra.org)^17^, (5) FRRP: http://frrp.org/data/^18^, (6) Safaie*et al*.^21^, and (7) Kumagai *et al*.^22^ (Fig. 4). There are few data on coral bleaching before the 1998 bleaching event and most data were collected in 2015 and 2016 (Fig. 4). The database however has good spatial coverage with coral bleaching data for 14,405 sites in 93 countries (Fig. 2).

## Data Records

The GCBD is stored at figshare^23^. Below we describe 20 Tables (also see Fig. 3 schematic) that comprise the GCBD: (1) Site_Info_tbl, (2) Sample_Event_tbl, (3) R_Scripts_tbl, (4) Cover_tbl, (5) Bleaching_tbl, (6) Environmental_tbl, (7) Authors_LUT, (8) Bleaching_Level_LUT, (9) City_Town_Name_LUT, (10) Country_Name_LUT, (11) Data_Source_LUT, (12) Ecoregion_Name_LUT, (13) Exposure_LUT, (14) Ocean_Name_LUT, (15) Realm_Name_LUT, (16) State_Island_Province_Name_LUT, (17) Substrate_Type_LUT, (18) Relevant_Papers_tbl, (19) Severity_Code_LUT, and (20) Bleaching_Prevalence_Score_LUT, where LUT stands for look-up table.

1. Site Information (**Site_Info_tbl**)
  Latitude_Degrees: latitude coordinates in decimal degrees.
  Longitude_Degrees: longitude coordinates in decimal degrees.
  Ocean_Name: the ocean in which the sampling took place.
  Realm_Name: identification of realm as defined by the Marine Ecoregions of the World (MEOW)^12^.
  Ecoregion_Name: identification of the Ecoregions (150) as defined by Veron *et al*.^13^.
  Country_Name: the country where sampling took place.
  State_Island_Province_Name: the state, territory (e.g., Guam) or island group (e.g., Hawaiian Islands) where sampling took place.
  City_Town_Name: the region, city, or nearest town, where sampling took place.
  Site_Name: the accepted name of the site or the name given by the team that sampled the reef.
  Distance_to_Shore: the distance (m) of the sampling site from the nearest land.
  Exposure: a site was considered exposed if it had >20 km of fetch, if there were strong seasonal winds, or if the site faced the prevailing winds. Otherwise, the site was considered sheltered or ‘sometimes’. ‘Sometimes’ refers to a few sites with a >20 km fetch through a narrow geographic window, and therefore we considered that the site was potentially exposed during cyclone seasons. We left the category ‘sometimes’ in the database because those sites were not clearly exposed sites, nor were they clearly sheltered sites, and future researchers may be interested in temporary exposure.
  Turbidity: kd490 with a 100-km buffer.
  Cyclone_Frequency: number of cyclone events from 1964 to 2014.
  Comments: comments of any issues with the site or additional information.
2. Sample Event Information (**Sample_Event_tbl**)
  Site_ID: site ID field from Site_Info_tbl.
  Reef_ID: name of reef site that was adopted by sampling group (from ReefCheck).
  Quadrat_No: quadrat number (from McClanahan *et al*.)^20^.
  Date_Day: the date of the sampling event.
  Date_Month: the month of sampling event.
  Date_Year: the year of sampling event.
  Depth: depth (m) of sampling site. Comments: comments of any issue or additional information of sampling event.
3. R Code (**R_Scripts_tbl**)
  Relevant_Papers_ID: relevant papers ID field from Relevant_Papers_tbl.
  Project name: name of project associated with R code.
  Paper_Title: title of paper where R code was published.
  Code_Name: name of R code file.
  Description: description of the R code.
  Data_Source: data source ID field from Data_Source_LUT.
  R_Code: attachment of R code file.
  URL: hyperlink to R code or link to github.
4. Coral Cover Information (**Cover_tbl**)
  Sample_ID: sampled ID field from Sample_Event_tbl.
  Substrate_Type: substrate type ID field from Substrate_LUT.
  S1: Reef Check breaks down transects into four 20 m × 5 m segments, point data from segment one of transect.
  S2: Reef Check breaks down transects into four 20 m × 5 m segments, point data from segment two of transect.
  S3: Reef Check breaks down transects into four 20 m × 5 m segments, point data from segment three of transect.
  S4: Reef Check breaks down transects into four 20 m × 5 m segments, point data from segment four of transect.
  Perc_hardcoral: percent hard coral cover from McClanahan *et al*.^20^ data source.
  Perc_macroalgae: percent macroalgae cover from McClanahan *et al*.^20^ data source.
  Average_Ellipse_Transect: calculated percent hard coral cover per 10 m × 1 m transect using ellipse equation.
  Average_Ellipse_Site: calculated percent hard coral cover per site using ellipse equation.
  Comments: comments of any issue or additional information of sampling event
5. Bleaching Information (**Bleaching_tbl**)
  Sample_ID: sample ID field from Sample_Event_tbl.
  Bleaching_Level: Reef Check data, coral population or coral colony.
  S1: Reef Check breaks down transects into four 20 m × 5 m segments, percent bleaching from segment one of transect.
  S2: Reef Check breaks down transects into four 20 m × 5 m segments, percent bleaching from segment two of transect.
  S3: Reef Check breaks down transects into four 20 m × 5 m segments, percent bleaching from segment three of transect.
  S4: Reef Check breaks down transects into four 20 m × 5 m segments, percent bleaching from segment four of transect.
  Percent_Bleaching_RC_Old_Method: old method of determining percent bleaching from Reef_Check.
  Severity_Code: coded range of bleaching severity from Donner *et al*.^10^.
  Percent_Bleached: percent of coral bleaching.
  Number_Bleached_colonies: number of bleached corals from McClanahan *et al*.^20^ data source.
  Bleaching_intensity: from McClanahan *et al*.^20^ data source.
  Bleaching_Prevalence_Score: coded range of bleaching prevalence from Safaie *et al*.^21^.
6. Environmental Parameter Information (**Environmental_tbl**)
  Sample_ID: sample ID field from Sample_Event_tbl.
  **ClimSST: CoRTAD. [Climatological Sea-Surface Temperature (SST)]** based on weekly SSTs for the study time frame, created using a harmonics approach.
  Temperature_ Kelvin: CoRTAD. SST in Kelvin.
  Temperature_Mean: CoRTAD. Mean SST in degrees Celsius.
  Temperature_Minimum: CoRTAD. Minimum SST in degrees Celsius.
  Temperature_Maximum: CoRTAD. Maximum SST in degrees Celsius.
  Temperature_Kelvin_Standard_Deviation: CoRTAD. Standard deviation of SST in Kelvin.
  Windspeed: CoRTAD. meters per hour.
  **SSTA: CoRTAD. (Sea-Surface Temperature Anomaly)** weekly SST minus weekly climatological SST.
  SSTA_Standard_Deviation: CoRTAD. The Standard Deviation of weekly SSTA in degrees Celsius over the entire period.
  SSTA_Mean: CoRTAD. The mean SSTA in degrees Celsius over the entire period.
  SSTA_Minimum: CoRTAD. The minimum SSTA in degrees Celsius over the entire period.
  SSTA_Maximum: CoRTAD. The maximum SSTA in degrees Celsius over the entire period.
  SSTA_Frequency: CoRTAD. (Sea Surface Temperature Anomaly Frequency) number of times over the previous 52 weeks that SSTA >  = 1 degree Celsius.
  SSTA_Frequency_Standard_Deviation: CoRTAD. The standard deviation of SSTA Frequency in degrees Celsius over the entire time period of 40 years.
  SSTA_FrequencyMax: CoRTAD. The maximum SSTA Frequency in degrees Celsius over the entire time period.
  SSTA_FrequencyMean: CoRTAD. The mean SSTA Frequency in degrees Celsius over the entire time period of 40 years.
  **SSTA_DHW: CoRTAD. (Sea Surface Temperature Degree Heating Weeks)** sum of previous 12 weeks when SSTA >  = 1 degree Celsius.
  SSTA_DHW_Standard_Deviation: CoRTAD. The standard deviation SSTA DHW in degrees Celsius over the entire period.
  SSTA_DHWMax: CoRTAD. The maximum SSTA DHW in degrees Celsius over the entire time period of 40 years.
  SSTA_DHWMean: CoRTAD. The mean SSTA DHW in degrees Celsius over the entire time period of 40 years.
  **TSA: CoRTAD. (Thermal Stress Anomaly)** weekly SSTs minus the maximum of weekly climatological SSTs in degrees Celsius.
  TSA_Standard_Deviation: CoRTAD. The standard deviation of TSA in degrees Celsius over the entire time period of 40 years.
  TSA_Minimum: CoRTAD. The minimum TSA in degrees Celsius over the entire time period of 40 years.
  TSA_Maximum: CoRTAD. The maximum TSA in degrees Celsius over the entire time period of 40 years.
  TSA_Mean: CoRTAD. The mean TSA in degrees Celsius over the entire time period of 40 years.
  TSA_Frequency: CoRTAD. The number of times over previous 52 weeks that TSA >  = 1 degree Celsius.
  TSA_Frequency_Standard_Deviation: CoRTAD. The standard deviation of frequency of TSA in degrees Celsius over the entire time period of 40 years.
  TSA_FrequencyMax: CoRTAD. The maximum TSA frequency in degrees Celsius over the entire time period of 40 years.
  TSA_FrequencyMean: CoRTAD. The mean TSA frequency in degrees Celsius over the entire time period of 40 years.
  **TSA_DHW: CoRTAD. (Thermal Stress Anomaly Degree Heating Weeks)** sum of previous 12 weeks when TSA >  = 1 degree Celsius.
  TSA_DHW_Standard_Deviation: CoRTAD. The standard deviation of TSA DHW in degrees Celsius over the entire time period of 40 years.
  TSA_DHWMax: CoRTAD. The maximum TSA DHW in degrees Celsius over the entire time period of 40 years.
  TSA_DHWMean: CoRTAD. The mean TSA DHW in degrees Celsius over the entire time period of 40 years.
7. Author Names (**Authors_LUT**)
  Last_Name: author’s last name.
  First_Name: author’s first name.
  Middle_Initial: author’s middle initial.
8. Bleaching Level Information (**Bleaching_Level_LUT**)
  Bleaching_Level: Reef Check data, coral population or coral colony.
9. City, Town Names (**City_Town_Name_LUT**)
  City_Town_Name: the region, city, or town, where sampling took place.
10. Country names (**Country_Name_LUT**)
  Country_Name: name of the country where sampling took place.
11. Data Source Information (**Data_Source_LUT**)
  Data_Source: name of source of original data set.
  Sample_Method: Description of the sampling methods used to collect the data. If more than one method was used then we stated that an amalgamation of methods were used to collect the data, and the original papers are found in “Relevant_Papers_tbl”, and can be referenced therein.
12. Ecoregion Names (**Ecoregion_Name_LUT**)
  Ecoregion_Name: name of Ecoregion from Veron *et al*.^13^.
13. Exposure Type (**Exposure_LUT**)
  Exposure_Type: site exposure to fetch.
14. Ocean Name Information (**Ocean_Name_LUT**)
  Ocean_Name: name of ocean where sampling took place.
15. Name of Realm (**Realm_Name_LUT**)
  Realm_Name: name of realm as identified by the Marine Ecoregions of the World (MEOW)^12^.
16. State, Island, Province Name (**State_Island_Province_Name_LUT**)
  State_Island_Province_Name, Name of the state, territory (e.g. Guam) or island group (e.g. Hawaiian Islands) where sampling took place.
17. Substrate Type (**Substrate_Type_LUT**)
  Substrate_Type: type of substrate from Reef Check data.
18. Relevant Publications (**Relevant_Papers_tbl**)
  Data_Source: source associated with publication.
  Author_ID: author ID field from Authors_LUT.
  Title: title of published work.
  Journal_Name: name of publication journal.
  Year_Published: year of publication.
  Volume: volume number of journal.
  Issue: issue number of journal.
  Pages: page range of publication.
  URL: hyperlink to publication.
  DOI: DOI number of publication.
  pdf: pdf attachment of publication.
19. Severity Index Code (**Severity_Code_LUT**)
  Severity_Code: coded range of bleaching severity from Donner *et al*.^10^.
20. Bleaching Prevalence Code (**Bleaching_Prevalence_Score_LUT**)

Bleaching_Prevalence_Score: coded range of bleaching prevalence from Safaie *et al*. ^21^.

## Database Queries

Fourteen summary queries have been created so researchers can easily extract the information they might need from the database and generate spreadsheets for data analysis. The queries are labelled sequentially. For example, a summary query has been generated that shows the sites, dates, mean coral cover, and mean bleaching, which is entitled “Query 1_Summary_Bleaching_Cover.” Some queries are necessary for the summary queries and are labelled subqueries.

## Technical Validation

The GCBD was curated by a Database Administrator (CK). No outside contributions are expected at this time. When coral bleaching datasets were added, there was a procedure to validate and standardize the site localities, including the following:

1. To ensure consistency in the naming of site localities, latitude and longitude coordinates were entered into Google Earth. The country, state/island/province, and city/town names were all cross-checked and verified.
2. All latitude and longitude coordinates were compared to ensure that a sampling event was not duplicated across multiple dataset sources.
3. Coordinate points were removed if: (i) they were erroneous (i.e., a coordinate point was negative when it should be positive), (ii) they occurred on land, or (iii) they were >1 km from a coral reef.
4. Environmental and site data were added to each site, which included reef site exposure, distance to land, mean turbidity, cyclone frequency, and a suite of sea-surface temperature metrics at the times of survey.

## Data Availability

All R code that was used in the GCBD are embedded in the database.
